# Curve progression following selective and nonselective spinal fusion for adolescent idiopathic scoliosis: are selective fusions stable?

**DOI:** 10.1007/s43390-024-00943-7

**Published:** 2024-08-19

**Authors:** Richard E. Campbell, Theodore Rudic, Alexander Hafey, Elizabeth Driskill, Peter O. Newton, Harms Research Study Group, Keith R. Bachmann

**Affiliations:** 1 Department of Orthopedic Surgery, University of Virginia Health System, PO Box 800159, Charlottesville, VA 22908 USA; 2 Department of Orthopaedic Surgery, Cleveland Clinic, Cleveland, OH USA; 3 Department of Orthopaedic Surgery, Medical University of South Carolina, Charleston, SC USA; 4 Division of Orthopedics and Scoliosis, Rady Children’s Hospital, San Diego, CA USA

**Keywords:** Adolescent idiopathic scoliosis, Selective fusion, Linear models

## Abstract

**Purpose:**

The purpose of this study is to compare postoperative outcomes between selective and non-selective fusions longitudinally over the first five postoperative years.

**Methods:**

Patient parameters were retrieved from a multicenter, prospective, database. Patients with Lenke 1–6, B and C deformities were included. Patients were stratified into 2 groups: selective fusion (SF), if the last instrumented vertebra (LIV) was at or cranial to the lumbar apex, or non-selective fusion (NSF). Differences in coronal and sagittal radiographic outcomes were assessed with generalized linear models (GLMs) at 1-, 2- and 5- year postoperative outcomes. Five-year postoperative categorical radiographic outcomes, flexibility, scoliosis research society scores (SRS), and reoperation rates were compared between groups. Matched cohorts were created for subgroup analysis.

**Results:**

416 (SF:261, NF:155) patients, including 353 females were included in this study. The mean preoperative thoracic and lumbar Cobb angles were 57.3 ± 8.9 and 45.3 ± 8.0, respectively. GLMs demonstrated greater postoperative coronal deformity in the SF group (*p* < 0.01); however, the difference between groups did not change overtime (*p* > 0.05) indicating a relatively stable postoperative deformity correction. The SF group had a greater incidence of lumbar Cobb ≥ 26 degrees (*p* < 0.01). The NSF group demonstrated worse forward and lateral flexibility at 5-year postoperative outcome (*p* < 0.05). There was no difference in postoperative SRS scores between the SF and NSF groups. Reoperation rates were similar between groups.

**Conclusion:**

Selective fusion results in greater coronal plane deformity; however, this deformity does not progress significantly over time compared to non-selective fusion. Selective spinal fusion may be a beneficial option for a larger subset of patients than previously identified.

**Level of evidence:**

III.

## Introduction

Posterior spinal fusion remains the mainstay treatment for adolescent idiopathic scoliosis (AIS). The selection of the lowest instrumented vertebra (LIV) remains a significant decision, balancing deformity correction with mobility preservation. More distal LIVs result in decreased spinal mobility [1–6], and may accelerate degeneration of the unfused levels [7]. Lonner et al. investigated risk factors degenerative disk disease 10 years after AIS fusion and found an LIV of L4 to be the highest risk factor [8]. However, a more cranial LIV may increase the risk for coronal imbalance, distal adding-on, and progression of the un-instrumented lumbar curve [9–11]. While the Lenke AIS classification provides guidelines for LIV selection, surgeons frequently deviate from these guidelines [12].

Short- and long-term lumbar curve correction after selective thoracic fusion has been observed in multiple studies [6, 7, 10, 13–15]. Singla et al*.* evaluated 2-year outcomes of spinal fusion in patients with Lenke 3 AIS and observed a reduction in the lumbar Cobb angle by 50% [10]. Ohashi et al*.* studied 10-year outcomes of spinal fusion in Lenke 1–4 AIS and reported that non-selective fusion led to greater correction of deformity but also greater loss of flexibility, which was associated with less favorable patient-reported outcomes [6]. Unfortunately, many of these studies examine small groups of patients, only report outcomes at limited time points, or don’t control for confounding variables. These limitations can be improved with robust patient databases, and advanced statistical methods.

In addition to the lumbar Cobb, the lumbosacral takeoff angle (LSTOA) has recently emerged as a useful radiographic parameter [16–18]. Compared to non-selective fusion, selective fusion results in less correction of the LSTOA; however, it is unknown if this correction remains stable over time.

To date, no studies have compared radiographic, clinical, and patient-reported longitudinal outcomes of selective and non-selective spinal fusion in a non-categorical fashion. This study aims to compare longitudinal radiographic outcomes and postoperative clinical outcomes between patients with selective fusions and non-selective fusions.

## Materials and methods

**Fig. 1:**
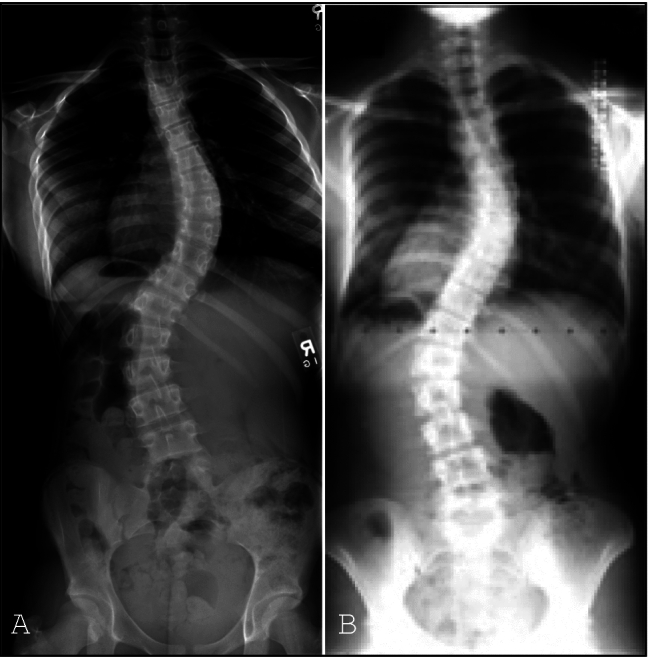
**A** Lenke 3 AIS with thoracic Cobb: 49, lumbar Cobb: 49, lumbar bend: 27, ATV ratio: 1.18. Underwent selective fusion to T11. **B** Lenke 6 AIS with thoracic Cobb: 47, lumbar Cobb: 48, lumbar bend: 28, ATV ratio: 1.06. Underwent non-selective fusion to L3

Patient information including demographics, clinical data, radiographic data, and normalized Scoliosis Research Society-22R (SRS-22R) scores was obtained from the AIS arm of the Harms Study Group database, a multicenter, prospective longitudinal database. This database contains data from 15 clinical sites. Consent for inclusion in this database and institutional review board approval was obtained prior to data collection. The database was queried to identify patients who underwent posterior spinal fusion for AIS with 5-year follow-up data. We wanted to include all patients with a large lumbar deformity that may be considered for selective fusion, regardless of Lenke classification. Although Lenke classification helps guide surgeon decision-making, there are some AIS deformities that may be technically Lenke 5 or 6 curves but are very similar to Lenke 3 or 4 curves (Fig. 1). Patients with preoperative thoracic Cobb angles < 45 were excluded. Patients with Lenke lumbar A modifier curves, or lumbar Cobb angles < 31 or > 60 were also excluded, as 99% of patients with NSFs had lumbar Cobb angles ≥ 31 degrees and 99% with SFs had lumbar Cobb angles ≤ 60. This created a more homogeneous cohort of patients that would potentially be considered for selective or non-selective fusion. Patients were stratified into 2 groups, selective fusion (SF) if the LIV was at or cranial to the lumbar apex vertebrae or non-selective fusions (NSF) if the LIV was caudal to the lumbar apex vertebrae. This classification was based on prior published definitions of selective fusion [16].

Data regarding reoperations at any time were queried from the database and reported for the entire cohort. The patient cohort then underwent analysis in four different manners based on available postoperative data. Only patients with all postoperative outcome data were included in each analysis. The first subgroup analysis consisted of a repeated measures longitudinal analysis of 1-, 2-, and 5-year postoperative radiographic data between groups. Outcome variables included postoperative thoracic Cobb angle, lumbar Cobb angle, LSTOA, C7 to central sacral vertical line absolute distance (C7-CSVL), thoracolumbar apical vertebral translation (AVT), thoracic kyphosis angle (T5-T12), lumbar lordosis angle (T12-S1), and sagittal vertical axis absolute distance (SVA). The LSTOA was measured as previously described [16]. The second cohort analysis compared 5-year categorical outcomes between groups. The outcome variables of interest are in Table 1. Subsequent matched subgroup analysis was performed for the first and second cohort. Patients were matched based on preoperative Risser score ± 1, thoracic Cobb angle ± 5 degrees, lumbar Cobb angle ± 5 degrees, and thoracic/thoracolumbar AVT ratio ± 0.75 and C7-CVSL ± 1 cm.

**Table 1:** Postoperative categorical radiographic outcomes

Parameter	Definition
Suboptimal postoperative lumbar Cobb	Postoperative lumbar Cobb ≥ 26 degrees [22]
Detrimental postoperative lumbar Cobb	Postoperative lumbar Cobb > 39 degrees [24]
Lumbar Cobb decompensation	Progression of the lumbar Cobb angle ≥ 10 degrees compared with 1st erect radiographs [19]
Coronal imbalance	Absolute value of C7-CSVL distance ≥ 2 cm [10, 19]
Progressive coronal imbalance	Increase in the absolute value of the C7-CSVL distance ≥ 5 mm compared with 1st erect radiographs
Adding-on	Change in disc angulation below the LIV > 5 degrees compared with 1st erect radiographs [18]
SVA imbalance	Absolute value of SVA ≥ 2 cm [31, 35]
Anterior SVA imbalance	SVA ≥ 2 cm
Posterior SVA imbalance	SVA ≤ -2 cm
Progressive SVA imbalance	Increase in the absolute value of the SVA ≥ 1 cm compared with 1st erect radiographs
5 yr proximal junctional kyphosis	A kyphotic angle > 10 degrees between the UIV and UIV + 1 levels and a kyphotic change of the same segment of > 10 degrees compared with preoperative radiographs
Progressive distal junctional kyphosis	> 10 degree increase in the angle between superior endplate of LIV and inferior endplate of LIV + 1 compared to 1st erect [36]
Shoulder imbalance	Shoulder height ≥ 2 cm compared to contralateral side [37, 38]
Trunk shift	Trunk shift > 2 cm from the CSVL [14, 38]

SVA: Sagittal vertical axis, CSVL: Central sacral vertical line

The third subgroup analysis compared preoperative, 5-year postoperative and delta (5-year postoperative − preoperative) SRS-22R scores between groups. The fourth subgroup analysis compared preoperative, 5-year postoperative and percent reduction in spine flexibility. Forward and lateral flexibility was manually measured by research personal as previously described [6]. Patients were also grouped into 4 groups based on LIV: T9–T12, L1–L2, L3, and L4–L5. Percent reduction in spine flexibility was compared between these LIV groups.

Statistical analysis was performed using SPSS version 28.0 (Armonk, NY: IBM Corp.). Chi-squared test and Fisher’s exact test were used to examine categorical data. One-way ANOVA and t tests were used to analyze parametric data. Kruskal–Wallis tests and Mann–Whitney U tests were used to examine nonparametric data. Generalized linear models were conducted to perform repeated measures longitudinal analyses of postoperative data. Covariates including age, gender, and preoperative outcome variable (e.g., preoperative LSTOA in LSTOA model) were controlled for in the models. Mean difference (MD) and 95% confidence intervals (CI) were reported when applicable. Angles were reported in degrees. Distances were reported in centimeters.

## Results

**Fig. 2:**
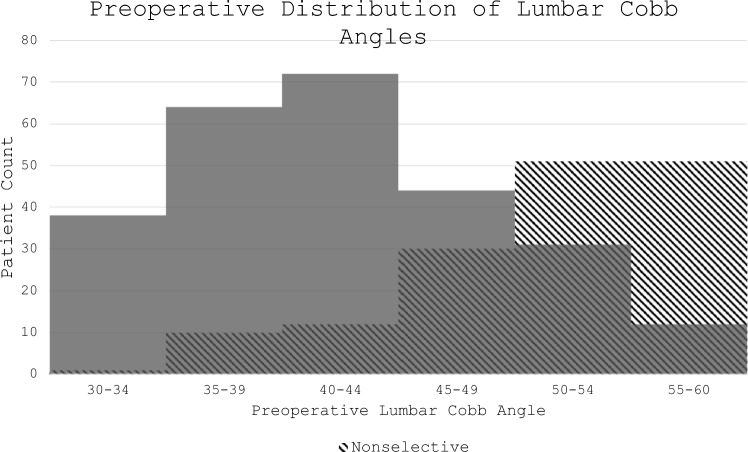
Distribution of preoperative lumbar Cobb angles in the selective and non-selective fusion groups

A total of 416 (SF:261, NSF:155) patients were included. The mean age at time of surgery was 14.5 ± 2.1 years. There were differences in the proportion of selective fusions between surgical institutions (*p*: 0.03). Preoperative parameters differed between groups; however, there was a large overlap in preoperative lumbar Cobb angles between groups (Fig. 2). Preoperative Lenke classifications are listed in Table 2. Twenty-four reoperations occurred in 20 patients. Single reoperation was due to pseudarthrosis in 2 patients, implant complications (broken implants, pedicle breeches, and prominent hardware) in 6 patients, infection in 5 patients, deformity progression in 3 patients and pseudoarthrosis with an implant complication in 1 patient. One patient underwent reoperations for infection and implant complications 3 years apart, another underwent 2 sequential reoperations for infection and concomitant implant complications, and another underwent 3 reoperations for infection over 2 years. In regard to deformity progression, one patient in the SF group underwent revision surgery for distal adding-on, and another for L1 screw cut-out resulting in distal kyphosis. One patient in the NSF group underwent reoperation for loose screws and L3-4 disk wedging. The mean time to reoperation was 2.7 ± 2.6 years (10 days to 7.8 years). The overall reoperation rate was similar between the NSF (5.2%) and SF group (4.6%) (*p*: 0.80). There were no differences in the rates of reoperations between groups when stratified by cause (Table 3).

**Table 2:** Preoperative Lenke classification

Preoperative Lenke classification	Selective fusion: 261	Nonselective fusion: 155
Lenke 1	147 (56.3%)	37 (23.9%)
Lenke 2	57 (21.8%)	14 (9.0%)
Lenke 3	40 (15.3%)	44 (28.4%)
Lenke 4	15 (5.7%)	15 (9.7%)
Lenke 5	0 (0%)	6 (3.9%)
Lenke 6	2 (0.8%)	39 (25.2%)

**Table 3:** Total cohort reoperations and associated diagnoses

	Nonselective fusion	Selective fusion	*p* value
	155 patients	261 patients	OR (95% CI)
	*N* (%)	*N* (%)	
Total reoperation	8 (5.2%)	12 (4.6%)	0.80
			0.9 (0.4–2.2)
Associated diagnosis			
Pseudoarthrosis	2 (1.3%)	2 (0.8%)	0.63
			0.4 (0.8–4.2)
Implant complication	2 (1.3%)	6 (2.3%)	0.72
			0.8 (0.3–2.0)
Infection	4 (2.6%)	4 (1.5%)	0.48
			0.6 (0.1–2.4)
Deformity progression	1 (0.6%)	2 (0.8%)	1.0
			1.2 (0.1–13.2)

Three patients had reoperations for multiple reasons

### 5-Year radiographic repeated measures analysis

**Fig. 3:**
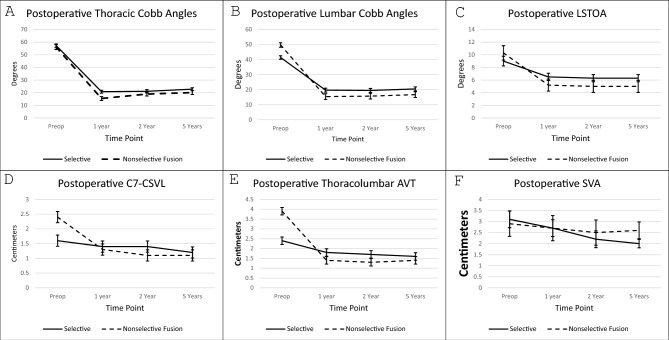
Five-year longitudinal radiographic outcomes following selective and non-selective spinal fusion including: **A** thoracic Cobb angle, **B** lumbar Cobb angle, **C** lumbosacral takeoff angle, **D** C7–CSVL, **E** thoracolumbar AVT, **F** absolute value sagittal vertical axis

A total of` 195 (SF:129, NSF:66) patients were included in the repeated measures analysis. The following longitudinal postoperative measurements were larger in the SF group compared to the NSF group, with no changes in the between-groups difference over time (group × time interaction): thoracic Cobb (*p* < 0.01), lumbar Cobb (*p* < 0.01), LSTOA (*p* < 0.01), absolute value C7-CSVL (*p* < 0.01), thoracolumbar apical translation (*p* < 0.01), thoracic kyphosis angles (p < 0.01). Lumbar lordosis angles were greater in the NSF group compared to the SF group; however, there was no change in the between-group difference over time (*p* < 0.01). Postoperative absolute value SVA measurements were similar between the SF and NSF groups (p: 0.24) (Fig. 3).

After matching, 70 (SF:35, NSF:35) patients were included in the subgroup analysis. Preoperative parameters were similar between groups (Table 4). The following longitudinal postoperative measurements were larger in the SF group than the NSF group, with no changes in the between-groups difference over time: thoracic Cobb (*p* < 0.01), lumbar Cobb angles (*p* < 0.01), LSTOA (*p* < 0.01), absolute value C7-CSVL distance (*p* < 0.01), thoracolumbar apical translation (*p* < 0.01), and thoracic kyphosis angles (*p* < 0.01). Lumbar lordosis angles were greater in the NSF group compared to the SF group; however, there was no change in the between-group difference over time (*p* < 0.01). Postoperative absolute values of SVA were similar (*p*: 0.30) between the SF and NSF groups over the first 5 postoperative years (Table 5)

**Table 4:** Total cohort preoperative patient characteristics

Preoperative parameter	Repeated measures analysis			Categorical outcomes analysis		
	NSF	SF	*p* value	NSF	SF	*p* value
	Full: 66; Matched: 35	Full: 129; Matched: 35		Full: 79; Matched: 38	Full: 133; Matched: 38	
Female Gender (%)						
Full cohort	59 (89.4%)	112 (86.8%)	0.61	71 (89.9%)	119 (89.5%)	0.93
Matched cohort	41 (83.7%)	44 (89.8%)	0.37	32 (94.1%)	29 (85.3%)	0.43
Open triradiate cartilage						
Full cohort	6 (9.1%)	19 (14.7%)	0.27	9 (11.4%)	23 (17.3%)	0.25
Matched cohort	1 (2.9%)	2 (4.1%)	1.0	1 (2.9%)	3 (8.8%)	0.61
Age (mean ± SD)						
Full cohort	14.2 ± 1.9	14.2 ± 2.0	0.92	14.2 ± 2.0	14.3 ± 2.0	0.81
Matched cohort	14.2 ± 1.6	14.4 ± 1.6	0.75	14.4 ± 1.7	14.4 ± 1.6	0.95
Thoracic scoliometer						
Full cohort	12.5 ± 5.2	14.7 ± 4.5	< 0.01	12.4 ± 5.4	14.5 ± 4.4	< 0.01
Matched cohort	13.9 ± 4.7	13.6 ± 4.6	0.74	13.3 ± 4.8	13.5 ± 4.1	0.85
Lumbar scoliometer						
Full cohort	9.8 ± 4.6	8.2 ± 4.2	0.02	9.9 ± 5.3	8.3 ± 4.4	0.03
Matched cohort	8.8 ± 4.0	10.0 ± 3.9	0.24	9.7 ± 5.0	9.0 ± 5.0	0.62
Thoracic Cobb (mean ± SD)						
Full cohort	56.1 ± 8.5	57.3 ± 8.3	0.26	56.3 ± 8.1	58.6 ± 9.1	0.06
Matched cohort	54.5 ± 7.6	55.5 ± 7.7	0.54	56.4 ± 9.1	56.9 ± 8.6	0.61
Lumbar Cobb (mean ± SD)						
Full cohort	49.7 ± 6.9	41.4 ± 6.4	< 0.01	50.2 ± 6.5	41.6 ± 6.1	< 0.01
Matched cohort	46.4 ± 6.7	45.1 ± 6.2	0.42	46.3 ± 6.3	44.4 ± 5.8	0.21
Thoracic/thoracolumbar AVT ratio						
Full cohort	1.3 ± 0.6	2.5 ± 1.4	< 0.01	1.3 ± 1.1	2.9 ± 2.8	< 0.01
Matched cohort	1.6 ± 0.6	1.6 ± 0.6	0.61	1.6 ± 0.6	1.8 ± 0.5	0.24
C7-CSVL absolute value						
Full cohort	2.4 ± 1.2	1.6 ± 1.1	< 0.01	2.3 ± 1.3	1.4 ± 0.9	< 0.01
Matched cohort	2.1 ± 1.1	1.7 ± 1.0	0.18	2.0 ± 1.0	1.8 ± 0.9	0.34
Lateral C7 to sacrum absolute value						
Full cohort	2.9 ± 2.2	3.1 ± 2.5	0.81	2.9 ± 2.2	3.0 ± 2.3	0.92
Matched cohort	2.7 ± 2.1	3.3 ± 2.5	0.32	3.1 ± 2.3	29 ± 2.2	0.71

SF, selective fusion; NSF, nonselective fusion; LIV, last instrumented vertebrae; LSTOA, lumbosacral takeoff angle; C7-CSVL, C7-central sacral vertical line; SD, standard deviation

.

**Table 5:** Repeated measures general linear models of selective and nonselective fusions

	Main cohort: SF: 129 NSF: 66			Matched cohort: SF: 35 NSF: 35		
	Mean difference (95% confidence interval (SF – NSF)	*p* value	Group × time interaction *p* value	Mean difference (95% confidence interval (SF – NSF)	*p* value	Group × time interaction *p* value
Thoracic Cobb angle	2.0 (0.9–3)	< 0.01	0.92	3.5 (1.8–5.1)	< 0.01	0.88
Lumbar Cobb angle	8.5 (7.1–9.9)	< 0.01	0.93	8.3 (6.5–10.2)	< 0.01	0.94
LSTOA	1.8 (1.2–2.3)	< 0.01	0.99	2.0 (1.1–2.9)	< 0.01	0.94
C7-CSVL absolute distance	0.3 (0.1–0.4)	< 0.01	0.54	0.4 (0.2–0.7)	< 0.01	0.89
Thoracolumbar AVT absolute distance	0.9 (0.7–1.0)	< 0.01	0.59	0.9 (0.6–1.1)	< 0.01	0.87
Thoracic ( T5-T12) Kyphosis	3.0 (1.8–4.4)	< 0.01	0.71	3.4 (1.0–5.7)	< 0.01	0.96
Lumbar Lordosis	− 2.7 (− 4.5 to − 0.9)	< 0.01	0.25	− 4.9 (− 7.9 to − 2.1)	< 0.01	0.40
SVA absolute distance	− 0.2 (− 0.6 to 0.1)	0.24	0.24	− 0.3 (1.0–0.3)	0.30	0.63

SF, selective fusion; NSF, nonselective fusion; LSTOA, lumbosacral takeoff angle; C7-CSVL, C7 to Central sacral vertical line; AVT, apical vertical translation; SVA, sagittal vertical axis

### 5-Year Postoperative Radiographic Categorical Outcomes

A total of 212 (SF:133, NSF:79) patients were included in the categorical analysis. At 5-year postoperative outcomes, the mean thoracic Cobb in the SF and NSF group was 56.3 ± 8.1 and 58.6 ± 9.1 (*p*: 0.06), respectively. The mean postoperative lumbar Cobb in the SF and NSF was 50.2 ± 6.5 and 41.6 ± 6.1 (*p* < 0.01), respectively. There was a greater incidence of lumbar decompensation in the NSF group compared to the SF group (*p*: 0.02). There were no other differences between groups.

After matching, 84 (SF:42, NSF:42) patients were included in the subgroup analysis. There was a higher incidence of postoperative lumbar Cobb angles ≥ 26 (p < 0.01) in the SF group compared to the NSF group (Table 6).

**Table 6:** Categorical radiographic outcomes of selective and nonselective fusions

	Main cohort					Matched cohort				
	NSF: 79		SF: 133		*p* value	NSF: 42		SF: 42		*p* value
	Count	%	Count	%	OR (95% CI)	Count	%	Count	%	OR (95% CI)
Suboptimal postoperative lumbar Cobb	10	12.7%	29	21.8%	0.10	3	8.8%	16	47.1%	** < 0.01**
					1.9 (0.9–4.2)					**9.2 (2.4–35.9)**
Detrimental postoperative lumbar Cobb	0	0%	1	0.8%	1.0	0	0%	0	0%	
Lumbar Cobb decompensation	7	8.9%	2	1.5%	**0.02**	2	5.9%	0	0%	0.49
					**0.2 (0.0–0.8)**					
Coronal imbalance	10	12.7%	21	15.8%	0.53	6	17.6%	9	26.5%	0.38
					1.3 (0.6–2.9)					1.7 (0.5–5.4)
Progressive coronal imbalance	14	17.7%	24	18.0%	0.96	9	26.5%	9	26.5%	1.0
					1.0 (0.5–2.1)					1.0 (0.3–2.9)
Adding-on	4	5.1%	17	12.8%	0.07	1	2.9%	3	8.8%	0.61
					2.7 (0.9–8.5)					3.1 (0.3–32.4)
SVA imbalance	42	53.2%	61	45.9%	0.30	18	52.9%	15	44.1%	0.47
					0.7 (0.4–1.3)					0.7 (0.3–1.8)
Anterior SVA imbalance	13	16.5%	17	12.8%	0.46	4	11.8%	5	14.7%	1.0
					0.7 (0.3–1.6)					1.3 (0.3–5.3)
Posterior SVA imbalance	29	36.7%	44	33.1%	0.59	14	41.2%	10	29.4%	0.31
					0.9 (0.5–1.5)					0.6 (0.2–1.6)
Progressive SVA imbalance	25	31.6%	36	27.1%	0.48	11	32.4%	8	23.5%	0.42
					1.2 (0.7–2.3)					1.5 (0.5–4.5)
PJK	7	8.9%	8	6.0%	0.44	3	8.8%	3	8.8%	1.0
					0.7 (0.2–1.9)					1.0 (0.2–5.3)
Progressive DJK	3	3.8%	1	0.8%	0.15	2	5.9%	0	0%	0.49
					0.2 (0.0–1.9)					
Shoulder imbalance	4	5.1%	7	5.3%	1.0	1	2.9%	2	5.9%	1.0
					1.0 (0.3–3.7)					2.0 (0.2–23.8)
Trunk shift > 2 cm	5	6.3%	13	9.8%	0.38	1	2.9%	5	14.7%	0.20
					1.6 (0.5–4.7)					5.7 (0.6–51.6)

Variables with p value <0.05 marked in bold

SF: selective fusion, NSF: nonselective fusion, SVA: PJK: DJK: distal junctional kyphosis, OR: odd ratio, CI: confidence interval

### 5-Year SRS patient-reported outcomes

A total of 273 (SF:169, NSF:104) patients were included in the SRS-22R analysis. There was no difference in SRS-22R scores between the SF and NSF groups at 5 years postoperatively, nor difference in delta (postoperative − preoperative) SRS-22R scores between the SF and NSF groups (Table 7).

**Table 7:** 5-year Postoperative Scoliosis Research Society -22R Scores

SRS-22R sub-scores	Preop mean score		*p* value	5-year postop mean score		*p* value	5-year postop mean change in score		*p* value
	NSF: 104	SF: 169		NSF: 104	SF: 169		NSF: 104	SF: 169	
Pain	4.0 ± 0.7	4.0 ± 0.8	0.82	4.2 ± 0.6	4.2 ± 0.7	0.54	0.2 ± 0.8	0.2 ± 0.9	0.71
Self-image	3.3 ± 0.6	3.4 ± 0.7	0.70	4.3 ± 0.6	4.3 ± 0.6	0.39	1.0 ± 0.7	1.0 ± 0.8	0.90
General function	4.4 ± 0.5	4.4 ± 0.6	0.92	4.4 ± 0.5	4.5 ± 0.4	0.57	0.0 ± 0.6	0.1 ± 0.6	0.37
Mental health	3.9 ± 0.7	4.0 ± 0.7	0.15	4.0 ± 0.7	4.0 ± 0.7	0.65	0.0 ± 0.9	0.0 ± 0.9	0.64
Satisfaction	3.6 ± 1.0	3.8 ± 0.9	0.10	4.5 ± 0.7	4.5 ± 0.7	0.94	0.9 ± 1.0	0.7 ± 1.1	0.13
SRS-22R total	3.9 ± 0.4	3.9 ± 0.5	0.51	4.3 ± 0.4	4.3 ± 0.5	0.82	0.4 ± 0.6	0.4 ± 0.6	0.73

SRS, scoliosis research society; SF, thoracic last instrumented vertebrae; NSF, lumbar last instrumented vertebrae; SD, standard deviation

### 5-Year clinical flexibility outcomes

**Fig. 4:**
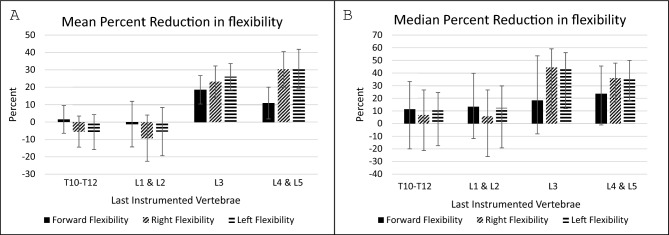
Five-year mean (**A**) and median (**B**) postoperative reduction in flexibility stratified by lowest instrumented vertebrae group

A total of 371 (SF:237, NSF:134) patients were included in the clinical flexibility analysis. At 5-year postoperative outcome, the SF group had greater forward flexibility (*p*: 0.02), left lateral flexibility (*p* < 0.01) and right lateral flexibility (*p* < 0.01) (Table 8). When stratified by LIV, there was an association between a more caudal LIV and greater, right, and left flexibility reduction (Table 9, Fig. 4).

**Table 8:** 5-year postoperative flexibility

Clinical flexibility	NSF: 134	SF: 237	p-value	NSF: 134	SF: 237	p-value	NSF: 134	SF: 237	p-value
	Preop centimeters Mean ± SD			Postop mean centimeters Mean ± SD			5 Year mean percent reduction ± SD		
Forward flexion	10.4 ± 3.6	10.0 ± 3.3	0.36	8.0 ± 3.7	8.9 ± 3.3	0.02	14.8 ± 50.3	1.2 ± 59.7	0.02
Right flexion	16.0 ± 5.2	14.6 ± 5.5	0.02	10.2 ± 3.2	13.9 ± 5.9	< 0.01	27.6 ± 39.5	− 8.1 ± 63.4	< 0.01
Left flexion	16.1 ± 4.9	15.1 ± 5.6	0.09	10.4 ± 3.4	14.0 ± 5.9	< 0.01	28.4 ± 35.7	− 5.8 ± 68.1	< 0.01

SF, thoracic last instrumented vertebrae; NSF, lumbar last instrumented vertebrae; SD, standard deviation

**Table 9:** Flexibility outcomes by lowest instrumented vertebrae

LIV group	Spine flexibility in centimeters			LIV comparisons for left and right flexibility			
	Percent reduction in forward flexion	Percent reduction in right flexion	Percent reduction in left flexion	T10-T12	L1 and L2	L3	L4 and L5
	(Mean ± SD)	(Mean ± SD)	(Mean ± SD)	*p* value*	*p* value*	*p* value*	*p* value*
T10-T12: 126	1.6% ± 48.0	–5.5% ± 53.2	–5.9% ± 59.9	–	1.0	< 0.01	< 0.01
L1 and L2: 108	− 1.2% ± 71.2	–9.3% ± 72.5%	–5.5% ± 76.1	–	–	< 0.01	< 0.01
L3: 92	18.5% ± 40.9	23.1% ± 46.4	26.0% ± 38.2	–	–	–	1.0
L4 and L5: 45	10.9% ± 65.1	30.2% ± 36.5	30.6% ± 39.2	–	–	–	–

LIV, lowest instrumented vertebrae; SD, standard deviation

*Bonferroni adjusted *p* value

## Discussion

Determining the ideal LIV in spinal fusion remains a challenging task for surgeons. Our matched and nonmatched results demonstrate that selective fusion preserved more mobility at the expense of greater residual lumbar coronal deformity; however, the extent of deformity did not worsen over time compared to non-selective fusions, indicating that the stability of deformity correction is not substantially impacted by selective vs non-selective fusion. Although patients with selective fusions have more residual deformity, patient-reported outcomes are the same and reoperation for deformity progression is rare. While further investigation is needed, patients with more ‘lumbar dominant’ deformities, (Lenke 3s and 4s) that commonly undergo non-selective fusion, may be amenable to selective fusion.

Residual coronal lumbar deformity after selective fusion has been studied extensively, with multiple studies demonstrating over 40% correction in the lumbar Cobb angle at 2-year follow-up [10, 16, 19, 20]. Bachmann et al*.* reported improvement in the LSTOA and lumbar Cobb angle in both NSFs and SF for Lenke 1 and 3 AIS, with larger correction after non-selective fusion [16]. Similarly, Singla et al*.* reported 68% reduction in the lumbar Cobb angle 2 years after fusion at or caudal to L3 of Lenke 3 AIS curves, compared to a 52% reduction with a more cranial LIV [10]. Long-term reduction in the lumbar Cobb angle has also been observed after selective thoracic fusion, corroborating our results that indicate lumbar deformity doesn’t worsen overtime comparted to non-selective fusions [14, 15, 21]. We did identify a greater incidence of postoperative lumbar Cobb angles ≥ 26 with SFs; however, the clinical implication of this is unknown. This cutoff, published by Schulz et al*.*, was based on postoperative confidence intervals and surgeon opinion [22]. While it was associated with patient satisfaction, it has not been associated with worse objective outcomes such as disk degeneration. Greater postoperative lumber Cobb angles are associated with lumbar disk degeneration; however, a reliable specific threshold has not been identified [23]. Akazawa et al. investigated lumbar spine degenerative changes on MRI in middle-aged AIS patients that were treated nonoperatively. These patients had a mean age of 45.6 (36–63) years and a mean lumbar curve of 48.6 degrees. The authors found that a lumbar Cobb angle threshold of 39.5 was 79% sensitive and 64% specific for Modic changes associated with disk degeneration on MRI [24]. However, it is important to note that this was identified in patients treated nonoperatively.

Postoperative coronal imbalance after selective fusion is also a concern. Kwan et al. reported 21% coronal imbalance at 2-year follow-up, and Larson et al*.* reported 43% and 29% coronal imbalance at 5- and 20-year follow-ups, respectively [14, 19]. In the present study, postoperative C7-CSVL distance was greater in the SF group. However, the incidence of coronal imbalance was similar between groups, including the matched cohort analysis. Furthermore, postoperative progression of coronal imbalance was similar between patients with selective and non-selective fusion. There were other radiographic differences between the SF and NSF groups, such as thoracic kyphosis and lumbar lordosis; however, these differences were small and likely clinically insignificant.

There was a trend toward a greater incidence of adding-on in the SF group compared to the NSF group, with 12.8% and 5.1% in the SF and NSF groups, respectively. These values are comparable to previously published rates [9, 19, 25]. Adding-on is typically thought of as a postoperative phenomenon after selective fusions, so the greater incidence compared with non-selective fusions is hard to clinically interpret. There was a greater rate of lumbar Cobb decompensation in the NSF group that may represent a similar phenomenon or may also be related to leaving too much rotation at the distal end of the lumbar curve. Notably, only one patient (0.3% of selective fusions) underwent revision for adding-on, and there was no difference in the incidence of revision for implant complications, or pseudoarthrosis. Prior studies have identified a higher rate of reoperation with an LIV at or distal to L4 at 40 years postoperative [26]. The lack of differences in reoperation rates in the current study may be due to the length of follow-up.

Our results demonstrated greater postoperative flexibility with selective fusion. Flexibility was significantly decreased as the fusion was extended caudally. Less spinal flexibility has been correlated with increased pain and decreased physical function at long-term follow-up [2, 4]. Ohashi et al*.* observed lower SRS scores in patients who experienced ≥ 40% postoperative reduction in lateral flexion, but found no difference in SRS scores between SF and NSF groups, despite reduction rates of ≥ 40% being three times more common in the NSF group [6]. Similarly, our study did not demonstrate a difference in SRS scores between groups. However, Sanchez-Raya et al*.* identified a relationship between fusion distal to L3 and worse SRS subtotal and pain scores [4]. Ahonen et al*.* reported better postoperative SRS-24R pain and satisfaction and total scores in patients with an LIV at or cranial to L2, compared to L3 or caudal [27]. However, prior studies have also reported worse patient-reported self-image scores with selective fusion or greater postoperative lumbar Cobb angles [28–30]. Several other studies have demonstrated no difference in SRS scores between selective and non-selective fusions [3, 10, 31]. The lack of consensus may be attributable to the SRS questionnaire itself, which has demonstrated high rates of ceiling effects [32]. It is also possible that there is no difference in this early postoperative period, but that differences may arise with longer follow-up.

The strengths of our study include a large sample size and statistical power. The generalizability of our findings is bolstered by the inclusion of patients from multiple centers and a wide range of AIS curves. Our large sample of patients with individual data at multiple postoperative time-points allowed for the use of statistical linear models, which enabled us to control for confounding variables, examine trends in relationships over time and decrease the chance of type 1 error when analyzing multiple outcome variables at several time points. This study has several limitations. Most of the outcomes are limited to the first 5 postoperative years. The heterogeneity of our sample population may have decreased internal validity. Certain surgical institutions were more likely to perform selective fusions and different surgeons may achieve different amounts of correction with selective fusion. The PJK definition used was slightly different than prior definitions due to availability of data. However, we believe this data is still reflective of overall trends [33, 34]. Clinical flexibility in the database was measured manually and may have been affected by patient effort. Similarly, radiographic outcomes were limited to plain radiographs; imaging such as MRIs were not available for review.

In conclusion, during the first 5 postoperative years, patients with AIS who underwent selective spinal fusion demonstrated greater postoperative flexibility, at the cost of greater postoperative deformity; however, there was no progression of this deformity over time. Our short-term results indicated that selective spinal fusion may be indicated for a larger subset of patients than previously identified.

## Data Availability

All data obtained from the Harms Study Group multicenter international prospectively collected database.
